# Physiological and transcriptome analysis of *Magnolia denudata* leaf buds during long-term cold acclimation

**DOI:** 10.1186/s12870-021-03181-5

**Published:** 2021-10-08

**Authors:** Kunjing Wu, Xiaojing Duan, Zhonglong Zhu, Ziyang Sang, Jie Duan, Zhongkui Jia, Luyi Ma

**Affiliations:** 1 Beijing Advanced Innovation Center for Tree Breeding by Molecular Design, College of Biological Sciences and Technology, Beijing Forestry University, Beijing, 100083 People’s Republic of China; 2 National Energy R&D Center for Non-food Biomass, Beijing Forestry University, Beijing, 100083 People’s Republic of China; 3 Forestry Science Research Institute of Wufeng County, Wufeng, 443400 Hubei Province China; 4 College of Forestry, Engineering Technology Research Center of Pinus tabuliformis of National Forestry and Grassland Administration, Beijing Forestry University, Beijing, 100083 China

**Keywords:** Cold acclimation, *Magnolia denudata*, Transcriptome, Signaling pathway

## Abstract

**Background:**

*Magonlia denudata* is an important perennial tree species of the Magnoliaceae family, known for its ornamental value, resistance to smoke pollution and wind, role in air purification, and robust cold tolerance. In this study, a high-throughput transcriptome analysis of leaf buds was performed, and gene expression following artificial acclimation 22 °C, 4 °C and 0 °C, was compared by RNA sequencing.

**Results:**

Over 426 million clean reads were produced from three libraries (22 °C, 4 °C and 0 °C). A total of 74,503 non-redundant unigenes were generated, with an average length of 1173.7 bp (N50 = 1548). Based on transcriptional results, 357 and 235 unigenes were identified as being upregulated and downregulated under cold stress conditions, respectively. Differentially expressed genes were annotated using Gene Ontology and the Kyoto Encyclopedia of Genes and Genomes pathway analyses. The transcriptomic analysis focused on carbon metabolism and plant hormone signal transduction associated with cold acclimation. Transcription factors such as those in the basic helix-loop-helix and AP2/ERF families were found to play an important role in *M. denudata* cold acclimation.

**Conclusion:**

*M. denudata* exhibits responses to non-freezing cold temperature (4 °C) to increase its cold tolerance. Cold resistance was further strengthened with cold acclimation under freezing conditions (0 °C). Cold tolerance genes, and cold signaling transcriptional pathways, and potential functional key components for the regulation of the cold response were identified in *M. denudata*. These results provide a basis for further studies, and the verification of key genes involved in cold acclimation responses in *M. denudata* lays a foundation for developing breeding programs for Magnoliaceae species.

**Supplementary Information:**

The online version contains supplementary material available at 10.1186/s12870-021-03181-5.

## Background

*Magnolia denudata*, an important species in the Magnoliaceae family, is well-known for its ornamental value, resistance to smoke pollution and wind, and contribution to air purification. As a robust deciduous species thriving under climatic conditions, its origins are attributed to the southeastern region of North America, and is now cultivated in China, in areas south of the Yangtze River and in northern cities such as Beijing and Lanzhou in cold areas [1]. *M. denudata* can survive low or freezing temperatures conditions and regrow in the spring. This species demonstrates high cold tolerance and can be cultivated in northern China [2]. To understand the mechanisms by which *M. denudata* adapts to cold stress, it is important to investigate its cold-resistance mechanism.

Cold is considered a major abiotic stress factor that tends to negatively affect growth, development, reproduction, yield, and survival [3]. To reduce the adverse effects of cold stress, temperate -plants have evolved various adaptive mechanisms including cold acclimation a process in which plants improve freezing resistance after exposure to short days and low non-freezing temperatures for a specific period [4]. Cold acclimation is an important strategy for plants to survive cold winters and involves physiological, metabolic, and cellular changes occurring after sensing cold temperatures [5, 6]. Responses include cold signal transmission, alterations in cellular structure and lipid composition, increase in antioxidant levels, overexpression of cold-related transcription factors (TFs) and accumulation of osmoregulatory substances such as soluble sugar and protein [7–12]. Cold tolerance, especially freezing tolerance, can be markedly enhanced after cold acclimation [13].

Transcriptional regulation of gene expression plays an important role in plant adaptation and cold stress tolerance [14]; the C-repeat binding factor (CBF)/dehydration responsive element (DRE) plays a crucial role the transcriptional pathways related to cold tolerance. Cold-induced CBF is one of the members of the APETALA2/−ETHYLENE RESPONSE FACTOR (AP2/ERF) gene family, and can induce the expression of cold response (COR) genes by binding to cis-acting elements in their promoters [12]. Many genes belonging to COR, such as dehydrin, encode a wide range of cryoprotective proteins that confer protection to cells against cold-induced damage [15].

RNA sequencing (RNA-seq) has been widely used in the analysis of the tolerance response in plants such as *Elymus nutans* [16], *Camellia oleifera* [17], *Citrus paradisi* [18], and *Nicotiana tabacum* [19]. Sequencing *M. denudata* improves our understanding of the physical changes involved in cold tolerance, and provides an in-depth explanation of its remarkable cold-hardiness.

Therefore, it is imperative to further study the gene expression profile of *M. denudata* under cold acclimation conditions. However, few studies have investigated the metabolic pathways and TFs related to cold resistance mechanisms in this species. This study aims to bridge our understanding of the molecular mechanisms associated with phenotypic and physiological changes during artificial cold acclimation.

## Results

### Physiological responses of *M. denudata* to cold acclimation

To determine the ideal time for RNA-seq, we measured the physiological responses of *M. denudata* besides observing phenotypic changes. We chose five temperatures (22 °C, 12 °C, 4 °C, 0 °C, and − 4 °C) represented as N_1_ (control), CA_1_, CA_2_, CA_3_, and CA_4_, respectively, to observe the changes in *M. denudata* with decreasing temperature.

**Fig. 1:**
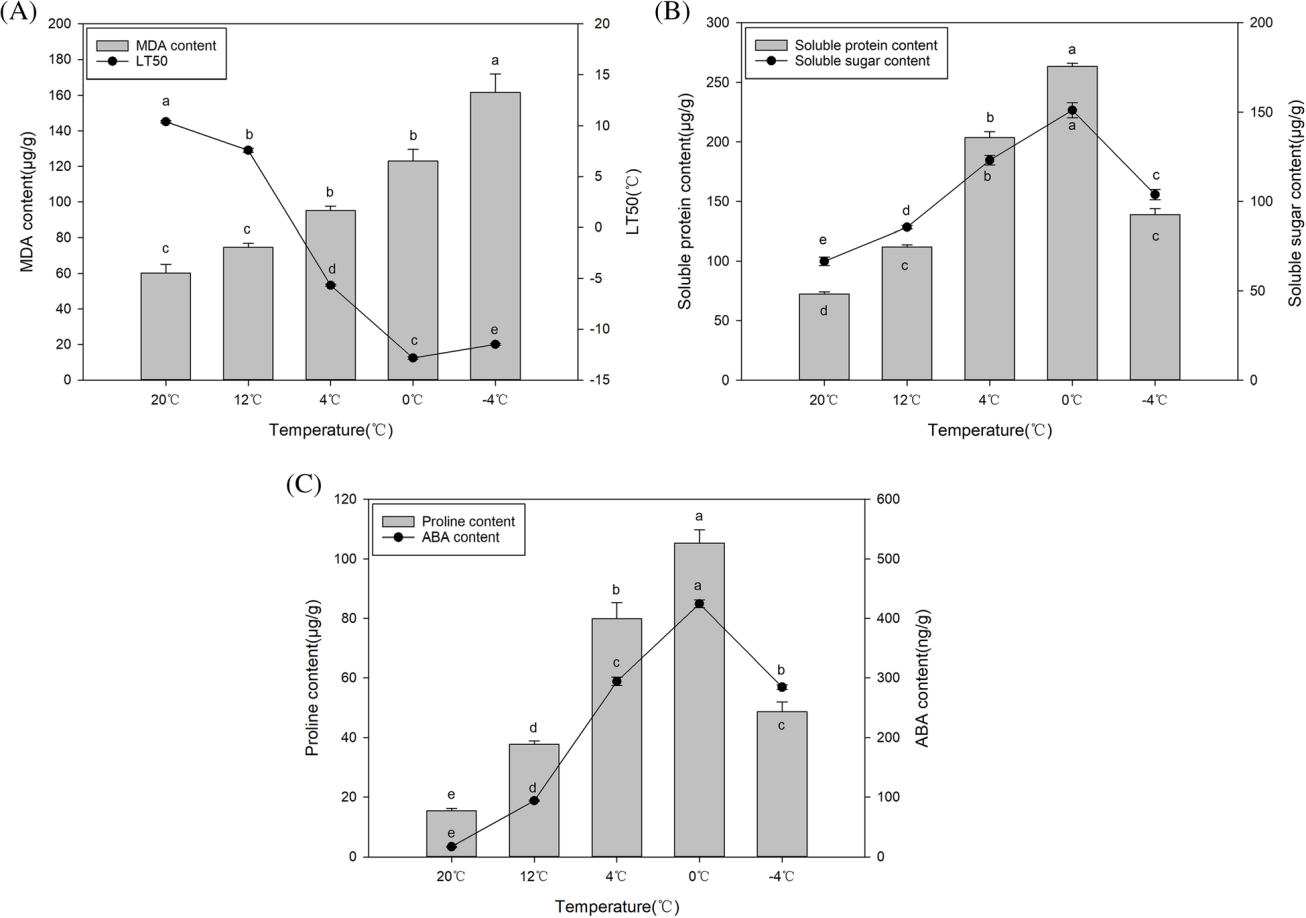
Physiological changes in leaf buds of *M. denudata* at different cold conditions. **A** MDA content, LT50, **B** soluble protein, soluble sugar content, **C** proline and ABA content in *M. denudata* at different temperatures. Lowercase letters indicate significant differences among different temperature treatments at *p* < 0.05

The median lethal temperature (LT50), obtained by determining the relative conductivity can reflect a plant’s cold resistance level [20]. Thus, we determined the LT50 and found that it was the highest at N_1_ (10.41 °C), followed by CA_1_ (7.59 °C), CA_2_ (− 5.68 °C), CA_3_ (− 12.83 °C), and CA_4_ (− 11.50 °C). Based on these results, cold acclimation is the strongest at 0 °C (Fig. 1A).

Malondialdehyde (MDA), as the final product of lipid peroxidation, is an important indicator for measuring oxidative damage under different stresses [21]. In contrast to LT50, the MDA concentration increased with the enhancement of the cold acclimation. At CA_1_, the MDA concentration of the shoot apex (buds) increased slightly and then increased significantly at CA_2_, followed by a gradual increase at CA_3_. The highest MDA concentration (nearly a two-fold increase) was obtained at CA_4_. The rate of increase in the MDA content of the buds was 23.76, 46.82, 65.95, and 82.51%, respectively, at CA_1_, CA_2_, CA_3_, and CA_4_. Based on the above values, the increase rate reached a maximum at − 4 °C (Fig. 1A).

Previous studies have shown that cold-resistance and other abiotic stresses in plants are associated with soluble sugar and soluble protein concentration [22]. With the extension of cold acclimation, the concentrations of soluble protein and soluble sugar increased from N_1_ to CA_3_, showing nearly a 4-fold and 2-fold increase, respectively, at CA_3_. However, the concentration decreased at CA_4_, with a value lower than that at CA_2_ (Fig. 1B). Hence, the maximum value was observed at CA_3_.

Similar to the above trend, the contents of proline and abscisic acid (ABA) showed slight increases at CA_1_, followed by sharp increases at CA_2_. Finally, the peak appeared at CA_3_ with a 7-fold and 25-fold increase for proline and abscisic acid, respectively. There was a steep decrease at CA_4_. Based on the above values, the maximum was observed at CA_3_ (Fig. 1C).

According to previous studies, 22 °C is the most suitable temperature for the growth of *M. denudata*; therefore in our study we set the control at 22 °C. In addition, 12 °C, which is a relatively warm temperature, did not show significant changes compared with the control. Based on our physiological data, at CA_4_, soluble sugar and protein concentrations decreased after 0 °C. At this time, we estimated that *M. denudata* had exceeded the limit of cold acclimation and suffered from freezing damage. In summary, we selected 22 °C (N_1_), 4 °C (CA_2_), and 0 °C (CA_3_) for the analysis of the transcriptome data to determine the molecular mechanisms underlying the cold acclimation of *M. denudata*.

### Transcriptome sequencing and de novo assembly

For the transcriptome assembly, a cDNA sample was prepared using high-quality RNA extracted from stem apexes (buds) for three libraries (4 °C [CA_2_], 0 °C [CA_3_], and control [N_1_]), and used for sequencing. Approximately 50 million reads were obtained for the control sample (N_1_), and 49 million raw reads were obtained for the two cold-treated samples (CA_2_ and CA_3_), respectively. The Q30 percentage (sequencing error rate < 1%) and the guanine and cytosine (GC) percentage were approximately 95 and 48% in the nine libraries, respectively (Table 1).

**Table 1:** Sequencing the *M. denudata* transcriptome in nine leaf buds samples from plants that controlled (N_11_, N_12_, N_13_) or cold-treated (CA_21_, CA_22_, CA_23_ and CA_31_, CA_32_, CA_33_)

Sample	Raw_Reads	Raw_Bases	Clean_Reads	Clean_Bases	Valid_Bases	Q30	GC
N_11_	49,865,558	7.48E+ 09	48,407,068	7.02E+ 09	93.85%	95.56%	47.17%
N_12_	49,758,436	7.46E+ 09	48,219,698	6.96E+ 09	93.29%	95.37%	47.25%
N_13_	49,744,998	7.46E+ 09	48,084,642	6.98E+ 09	93.52%	95.19%	47.17%
CA_21_	49,232,860	7.38E+ 09	47,539,238	6.86E+ 09	92.92%	94.80%	47.34%
CA_22_	49,135,554	7.37E+ 09	47,379,256	6.83E+ 09	92.69%	94.60%	48.25%
CA_23_	49,868,708	7.48E+ 09	48,208,074	6.92E+ 09	92.49%	94.36%	47.73%
CA_31_	49,536,752	7.43E+ 09	48,047,294	6.92E+ 09	93.07%	95.22%	48.44%
CA_32_	49,101,138	7.37E+ 09	47,295,846	6.82E+ 09	92.62%	94.62%	47.97%
CA_33_	44,909,042	6.74E+ 09	43,302,260	6.23E+ 09	92.55%	94.88%	48.01%

**Fig. 2:**
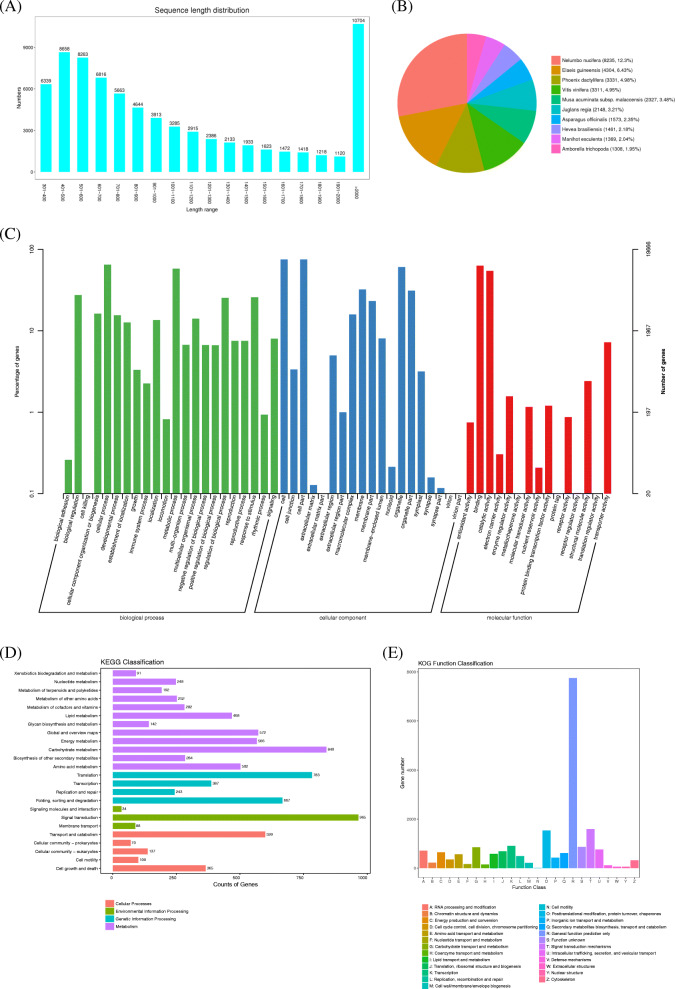
Characteristics and annotations of unigenes. **A** Unigene lengths distribution in *M. denudata* **B** Top 10 Species distribution for each unigene based on NR database **C** GO categorization functional annotation of the unigenes **D** KEGG annotation of the unigenes **E** Distribution of unigenes with KOG functional classification annotation

The de novo assembly yielded 74,503 unigenes with lengths ranging from 301 to 23,158 bp, and the average length was 1173.7 bp (Table 2). In general, the number of unigenes decreased with the increase of gene length after 400 bp; unigenes with a length of 401–500 bp (8658; 11.62%) accounted the largest proportion, followed by length from 501 bp to 600 bp (8263; 11.09%) (Fig. 2A).

**Table 2:** Statistics of transcriptome unigenes

Term	All(> 300 bp)	> = 500 bp	> = 1000 bp	N50	Total_Length	Max_Length	Min_Length	Average_Length
Unigene	74,503	59,612	30,238	1548	87,444,115	23,158	301	1173.7

### Annotation and classification of *M. denudata* unigenes

The assembled unigenes were searched against Nr, SwissProt, KEGG, KOG, eggNOG, GO, and Pfam. databases using BLASTx with an E-value < 10^− 5^. Among these unigenes, there were 31,803 and 19,666 exhibited significant differences (E-value <1e^− 5^) in the Nr and GO database, respectively. In addition, 22,385, 9403, 18,935,28,452, and 40 unigenes, respectively, were successfully annotated to known proteins in the other five databases (SwissProt, KEGG, KOG, eggNOG, and Pfam) (Table 3). In general, the unigene sequences were most similar to genes from *N. nucifera* (8235), followed by *E. guineensis* (4304), *P. dactylifera* (3331), *V. vinifera* (3311), and *M. acuminata subsp. malaccensis* (2327) via BLASTx matches (Fig. 2B).

**Table 3:** Annotation statistics of *M. denudata* unigenes

Anno_Database	Annotated_Number	300 < =length < 1000	length > =1000
NR	31,803 (42.69%)	10,988 (14.75%)	20,815 (27.94%)
Swissprot	22,385 (30.05%)	6461 (8.67%)	15,924 (21.37%)
KEGG	9403 (12.62%)	2813 (3.78%)	6590 (8.85%)
KOG	18,935 (25.42%)	6022 (8.08%)	12,913 (17.33%)
eggNOG	28,452 (38.19%)	9032 (12.12%)	19,420 (26.07%)
GO	19,666 (26.40%)	5596 (7.51%)	14,070 (18.89%)
Pfam	40 (0.05%)	30 (0.04%)	10 (0.01%)

### Gene annotation and functional classification of *M. denudata* unigenes

Gene Ontology (GO) was used to annotate the predicted *M. denudata* genes. According to the GO analysis, 19,666 unigenes were classified into the following three ontologies: biological process, cellular component, and molecular function. In the biological process category, genes involved in the ‘cellular process’ (12,765) and ‘metabolic process’ (11,346) were well represented. The cellular component category mainly comprised ‘cell’ (14,762), ‘cell parts’ (14,740), and ‘organelles’ (11,868). Within the molecular function category, ‘binding’ (12,358) and ‘catalytic activity’ (10,710) were highly represented, and there was a considerable number of genes pertaining to ‘biological regulation’ (5401), ‘membrane’ (6303), and ‘organelle part’ (6105) categories; however, few pertained to ‘cell killing’ (10), ‘virion’ (8), ‘virion part’ (6), and translation regulator activity (8) (Fig. 2C). The GO analysis identified a substantial number of genes related to biological processes and cellular components following cold acclimation.

The Kyoto Encyclopedia of Genes and Genomes (KEGG) is a signal pathway database used for analyzing the metabolic pathways, utilities of biological systems and functions of genes. Based on a comparison against the KEGG database, 9403 unigenes (12.62%) were found to be significantly enriched in the database and were assigned to 214 KEGG pathways. The most represented pathways were ‘Environmental Information Processing−−Signal transduction’ (965 members), ‘Metabolism−−Carbohydrate metabolism’ (840 members), and ‘Genetic Information Processing−−Translation’ (783 members) (Fig. 2D).

In total, 18,935 unigenes were assigned to a KOG classification and divided into 25 specific categories (Fig. 2E). The largest group was ‘general function prediction only’ (7750), followed by ‘signal transduction mechanisms’ (1589), ‘posttranslational modification’ and ‘protein turnover, chaperones’ (1530). Only a few unigenes were assigned to the ‘nuclear structure’ (60), and ‘cell motility’ (57) ‘cell motility’ (11) categories.

### Gene changes among the different phases of cold acclimation

**Fig. 3:**
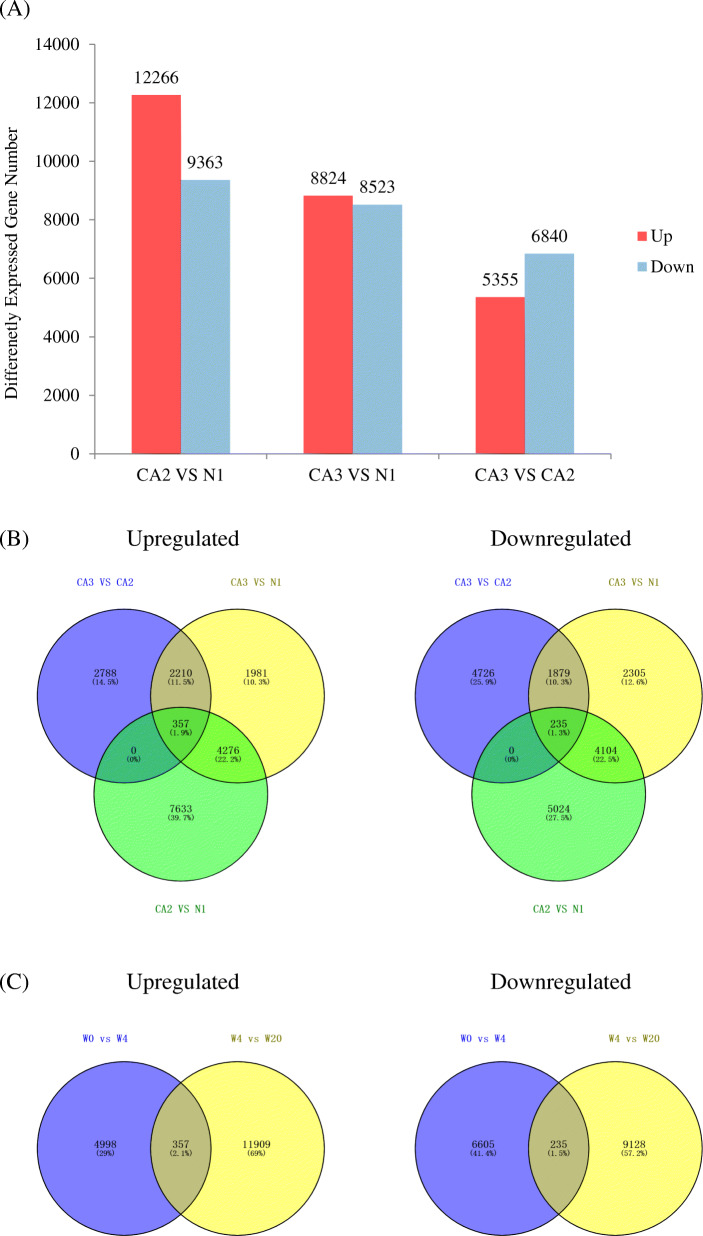
The expression of the gene changes in *M. denudata* during different cold acclimation stages. **A** Changes in gene expression profile during different cold acclimation stages. The numbers of up-regulated (red) and down-regulated (blue) genes between CA_2_ vs. N_1_, CA_3_ vs. N_1_ and CA_3_ vs. CA_2_ are summarized **B** Venn diagrams of CA_3_ vs. CA_2_, CA_3_ vs. N_1_ and CA_2_ vs. N_1_ differential genes expression **C** Venn diagrams of CA_3_ vs. CA_2_ and CA_2_ vs. N_1_ differential genes expression

Differentially expressed genes (DEGs) with a *p*-value < 0.05 and |log_2_FC| > 1 met the differential expression threshold. Among the DEGs identified, expression levels of 12,666 and 8824 genes were upregulated, and those of 9363 and 8523 genes were downregulated in the CA_2_ and CA_3_ groups, respectively. Between CA_3_ and CA_2_, 5355 up-regulated and 6840 down-regulated genes were identified; there were more DEGs in CA_2_ vs N_1_ than those observed in the other groups, while the number of DEGs in CA_3_ vs CA_2_ was the lowest of the three (Fig. 3A). Compared to N_1_, expression levels of 7633 (39.7%) genes were induced exclusively by CA_2_, while for CA_3_ only 1981 (10.3%) unigenes were specific. A similar trend in DEGs was observed in the repressed unigene groups with the development of cold acclimation; there were 5054 (27.5%) unigenes specifically expressed at CA_2_, while there were only 2305 (12.6%) unigenes expressed at CA_3_. Among these DEGs, 357 upregulated and 235 downregulated genes were detected in response to all treatments (Fig. 3B and C).

Overall, in *M. denudata*, transcript abundance changed significantly from N_1_ to CA_3_; the expression of cold response genes could be induced and expressed largely from N_1_ to CA_2_, although there were fewer changes in gene expression changes at CA_3_, these may be crucial to cold acclimation. At the same time, we found that there were less DEGs compared to CA_2_ vs. N_1_, while there were several DEGs showing expression in response to cold acclimation at CA_3_ which indicated the role CA_3_ played in cold acclimation.

### DEGs of assembled *M. denudata* transcripts under cold acclimation conditions

Compared to N_1_, GO analysis of DEGs at CA_2_ demonstrated that genes related to membrane structure and transcription were overexpressed. In biological process, cellular component, and molecular function, ‘transcription, DNA-templated’, ‘integral component of membrane’, and ‘DNA binding transcription factor activity’ were the most enriched GO categories. Compared to N_1_, for biological process, the major subcategory was ‘DNA integration’. In the cellular component, ‘plasma membrane’, and ‘extracellular region’ were the most representative subcategories, ‘nucleic acid binding’, ‘zinc ion binding’, and ‘aspartic-type endopeptidase activity’ were the top three subcategories in the ‘cellular component’ category in the development of cold acclimation at CA_3_ (Additional file 1: Table S1).

**Fig. 4:**
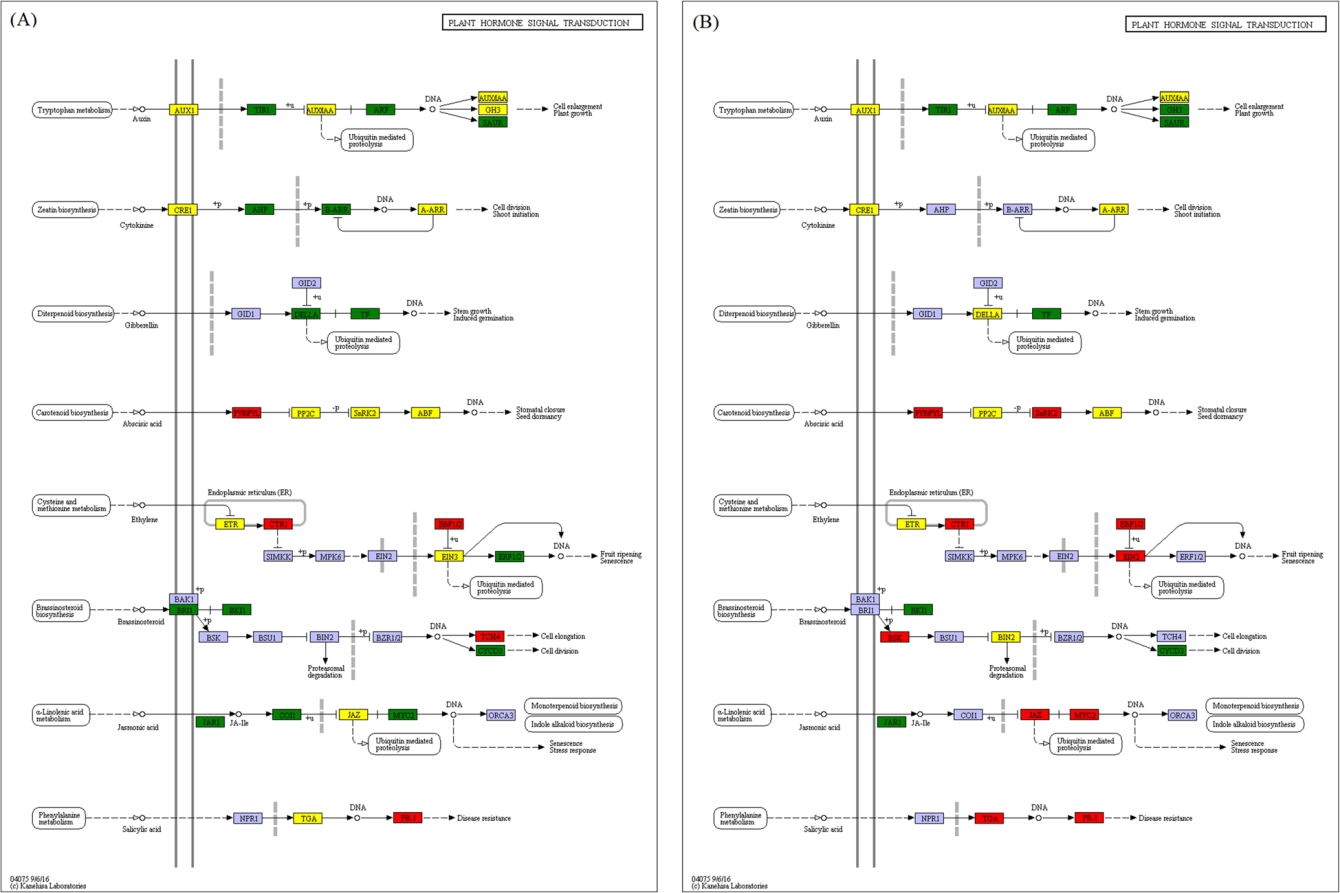
KEGG map about plant hormone signal transduction compared with N_1_. Red indicates significantly upregulated; green indicates significantly downregulated; yellow indicates proteins encoded by both up- and downregulated genes; purple indicates no significantly changed. **A** CA_2_ vs. N_1_; **B** CA_3_ vs. N_1_

Compared to N_1_, KEGG pathway enrichment analysis for DEGs indicated that three pathways, namely – ‘phenylpropanoid biosynthesis (ko00940)’, ‘biosynthesis of amino acids (ko01230)’ and ‘starch and sucrose metabolism (ko00500)’ were significantly enriched, and pathways associated with ‘plant hormone signal transduction (ko04075)’ (Fig. 4A), ‘starch and sucrose metabolism (ko00500)’ and ‘biosynthesis of amino acids (ko01230)’ were significantly decreased at CA_2_. At CA_3_ vs. N_1_, ‘carbon metabolism (ko01200)’, ‘protein processing in endoplasmic reticulum (ko04141)’, ‘starch and sucrose metabolism (ko00500)’, and ‘glycolysis / gluconeogenesis (ko00010)’ pathways were significantly enriched, and the ‘starch and sucrose metabolism (ko00500)’ and ‘plant hormone signal transduction (ko04075)’ (Fig. 4B) pathways were significantly depleted. Compared with CA_2_, ‘plant hormone signal transduction (ko04075)’ and ‘biosynthesis of amino acids (ko01230)’ pathways were enriched, ‘glycerophospholipid metabolism (ko00564)’, ‘glycerolipid metabolism (ko00561)’, and ‘DNA replication (ko03030)’ pathways were significantly depleted at CA_3_ (Additional file 2: Table S2).

### Response of important TFs and protein kinases (PKs) to cold acclimation

TF-regulated gene expression networks have an important effect on plant growth and development. To explore the TFs in response to cold acclimation in *M. denudata*, 261 TF genes (103 upregulated and 158 downregulated) were identified. These TFs were mainly concentrated in the basic helix-loop-helix (bHLH), ERF, WRKY, bZIP, GATA, MYB, zinc finger and AP_2_ families. Among all the genes evaluated, the number of genes for bHLH was the largest, with 52 genes (10 upregulated and 42 downregulated), followed by ERF with 27 genes (17 upregulated and 10 downregulated), WRKY with 22 genes (15 upregulated and 7 downregulated), bZIP with 17 genes (6 upregulated and 11 downregulated), GATA with 13 genes (6 upregulated and 7 downregulated), MYB with 13 genes (4 upregulated and 9 downregulated), zinc finger with 3 genes (1 upregulated and 2 downregulated), and AP_2_ with 3 genes (1 upregulated and 2 downregulated). The expression levels of the same gene was different for different periods of cold acclimation. The expression of CL6131Contig1 (the GT-3b gene) increased significantly at CA_2_ and then decreased significantly with the extension of treatment time (Additional file 3: Table S3).

A total of 423 DEGs encoding PKs were identified in the cold stress gene sets. There were 222 DEGs (86 upregulated and 136 downregulated) encoding serine/threonine-PKs (STPKs), including 60 DEGs encoding LRR receptor-like STPKs (18 upregulated and 42 downregulated), 33 DEGs encoding G-type lectin S-receptor-like STPKs (19 upregulated and 14 downregulated) and 7 DEGs encoding CBL-interacting STPK (5 upregulated and 2 downregulated). In addition, there were 16 DEGs (4 upregulated and 12 downregulated) encoding calcium-dependent PKs (CDPKs), 4 DEGs (3 upregulated and 1 downregulated) belonging to the CBL-interacting protein kinases (CIPKs) family, 7 DEGs (6 upregulated and 1 downregulated) belonging to the mitogen-activated protein kinase kinases (MAP 3Ks) family. We also found 91 receptor-like protein kinase (RLK) DEGs (36 upregulated and 49 downregulated) included in the group of STPKs, including 27 leucine-rich repeat RLKs, 14 cysteine-rich RLKs and 7 proline-rich RLKs. The expression of CL37311Contig1 and CL57668Contig1, which are inactive leucine-rich RLK and rust resistance kinase, increased at CA_2_ and then decreased at CA_3_ (Additional file 4: Table S4).

### Gene expression of biosynthesis and metabolism related to proline and hormones

Proline, as one of the most effective osmotic balance adjustment substances, accumulates rapidly when plants subjected to various abiotic stresses; 54 genes that are involved in the regulation of and relief from osmotic stress were involved in ‘arginine and proline metabolism (ko00330)’ based on the KEGG enrichment analysis (Additional file 7: Fig. S1). Expression levels of four genes (CL8828Contig1, CL12870Contig1, CL38215Contig1, and CL53606Contig1) for delta1-pyrroline-5-carboxylate synthetase (P5CS) were significantly upregulated. The expression of CL8828Contig1, CL12870Contig1, and CL53606Contig1 increased by 5-, 6.2-, and 12.9-fold, respectively, at CA_2_, and CL38215Contig1 increased by 3.2-fold at CA_3_ compared with CA_2_. DREB1, a key TF involved in proline synthesis, is highly expressed during cold acclimation and promotes proline synthesis for cold tolerance.

Expression of transportation inhibitor response 1 (TIR1) gene in the auxin signaling pathway was downregulated in CA_2_ and CA_3_ compared to the control. The auxin response factor and small auxin up RNA (SAUR) levels also decreased after CA_2_.

ABA plays a key role in the development of cold tolerance in plant cells. Since protein phosphatase 2C (PP2C) is a negative regulator of the ABA signaling pathway, pyrabactin resistance like (PYL) abscisic acid receptors bound to ABA can initiate ABA signaling by inhibiting PP2C activity. For example, expression levels of three DEGs (CL32501Contig1, CL32603Contig1, and comp14208_c0_seq1_2) encoding ABA receptor PYL family, were upregulated after CA_2_.

Ethylene, one of the five major types of plant hormones, plays a vital role in the development of resistance against the effect of cold. The expression levels of ethylene-insentive3 (EIN3)-binding F-box protein1/2 (EBF1/2) and constitutive triple response1 (CTR1), which are located downstream of the ethylene receptor, increased at CA_2_ (Additional file 5: Table S5).

### Validation of RNA-Seq by qRT-PCR

**Fig. 5:**
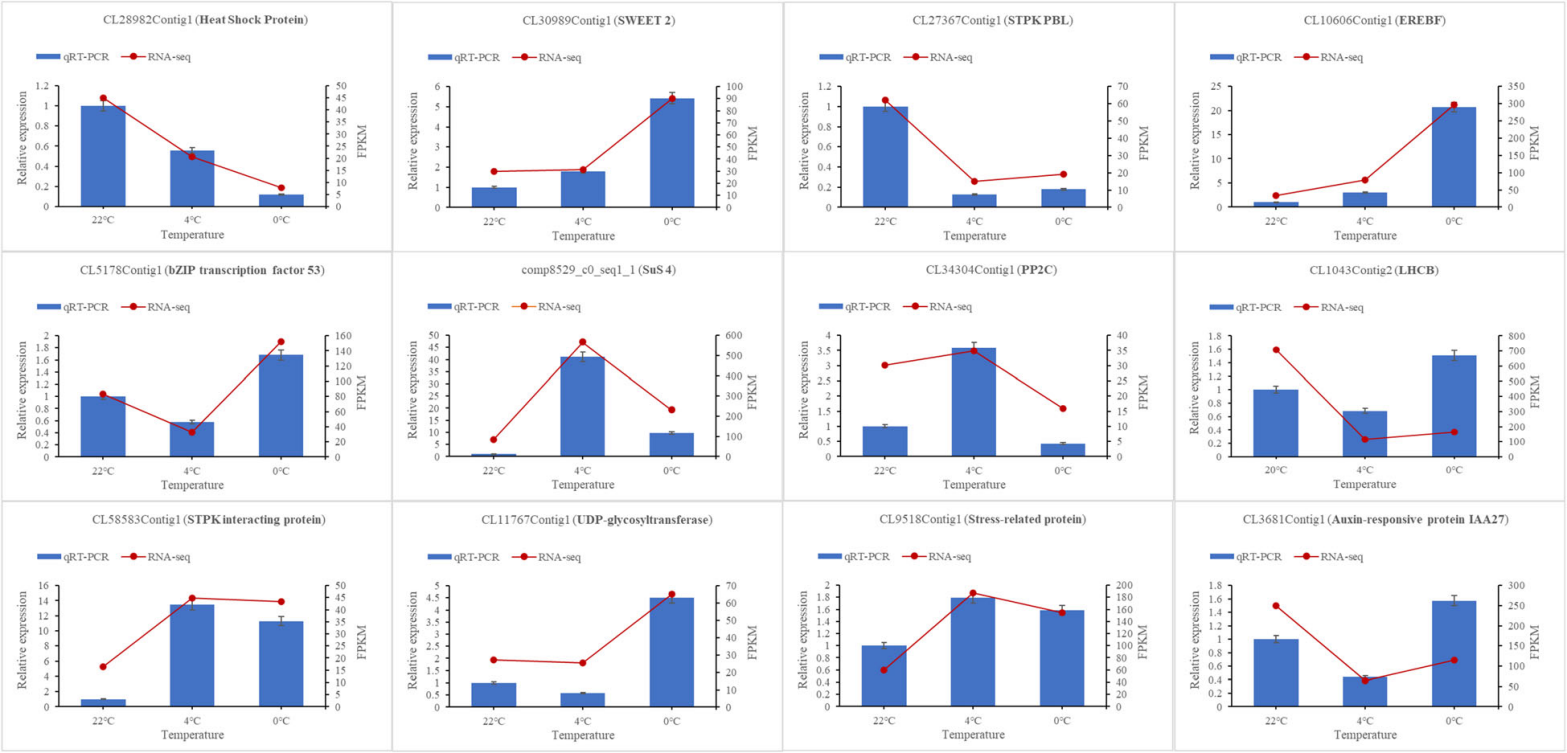
The expression of 12 DEGs from 22 °C(N_1_), 4 °C (CA_2_), 0 °C (CA_3_) and comparison between qRT-PCR and RNA-seq. The relative expression of the qRT-PCR assay is calculated by the 2^−ΔΔCT^ method and are displayed as blue columns; UBQ was used as the reference gene. FPKM values of RNA-seq are shown as red polylines

To validate the reliability of RNA-seq, 12 DEGs were selected and tested by qRT-PCR. The genes belonged to different categories including cold-responsive protein, sugar transport, PKs, metabolism, TFs and hormone-related signaling. The trends of genes under different treatments conditions observed using qRT-PCR were similar to those observed using RNA-seq, whereas two DEGs, the gene encoding light-harvesting complex II chlorophyll a/b binding protein 1 (LHCB) (CL1043Contig2) and auxin-responsive protein IAA27 (CL3681Contig1) displayed a slightly inconsistent expression pattern (Fig. 5), confirming the reliability of RNA-seq.

## Discussion

### Signaling mediates cold-stress responses

Cell membranes counter effects of low temperature with membrane rigidification after plants are subjected to cold stress, and subsequently the low-temperature signal is transmitted downstream through a secondary messenger such as Ca^2+^ or ABA. This triggers a Ca^2+^ influx, which acts upstream of the genes related to CBFs and COR [7, 12]. In this study, GO enrichment helped identify an important role of the ‘integral component of membrane’ in with the development of tolerance to cold stress, suggesting that membrane components were altered with the enhancement of the membrane fluidity. Under cold stress, the saturation of membrane lipid decreases with the upregulating content of fatty acid desaturase (FAD) which increases membrane fluidity, thereby increasing the cold tolerance [23, 24]. In our study, one FAD5 gene (CL30097Contig1) was identified whose expression downregulated at CA_2_ and upregulated at CA_3_ indicating a possible reason for the enhancement of cold resistance at 0 °C. Additionally, 31 DEGs related to calcium ion binding (15 upregulated and 16 downregulated) were identified, and only three of these DEGs were involved in the ABA-activated signaling pathway [25, 26]. In a previous study, Ca^2+^-dependent protein kinases (CDPKs) were verified as important sensors of Ca^2+^ flux in plants in response to different abiotic stresses [27], and have been shown to enhance abiotic stress tolerance [28]. In this study, 12 genes related to CDPK were identified. Notably, seven genes were downregulated from N_1_ to CA_2_ with the enhancement of cold resistance, indicating that genes related to CDPK were not completely functional at this juncture. Moreover, the expression of CL31785Contig1, a gene encoding CDPK, was first downregulated at CA_2_ and then upregulated at CA_3_. For *M. denudata*, the low temperature at CA_2_ did not completely stimulate the cold stress response, whereas extremely low temperatures such as those at CA_3_, could further strengthen cold tolerance. These results support the findings of this study, as soluble sugar content increased significantly at CA_2_ (cold) and CA_3_ (extreme cold conditions), and the stage from CA_2_ to CA_3_ was deemed for cold acclimation. However, in tomato (*Lycopersicon esculentum*) [29] and rice (*Oryza sativa*. L) [30], overexpression of CDPK induced the expression of genes related to the development of cold tolerance under cold stress conditions. This is not consistent with the results of this study, indicating a different function of CDPK in *M. denudata*.

Light signaling is also closely related to the exhibition of cold stress responses [31, 32]. Phytochromes are proved involving in cold tolerance by regulating CBF/DREB1s expression [33, 34]. Phytochrome interacting factor (PIFs), belonging to the bHLH family [35], can directly bind to phytochromes and transcriptionally regulate the expression level of the downstream genes of PHYA/B [36]. Several studies have revealed that *PIF1* [33], *PIF3* [37], and *PIF4* [32] exert negative effects on cold tolerance. In this study, *PIF1* (CL38596Contig1), *PIF3* (CL39360Contig1), and *PIF4* (CL38753Contig1) were identified, which were found to be downregulated at CA_2_ and CA_3_ compared with the control. Notably, expression of CL38753Contig1, which encoded *PIF4*, was first downregulated and then upregulated at CA_2_. Overall, temperatures below 4 °C had a negative effect on *M. denudata*.

### Hormone signaling and transport in response to cold acclimation

Plant hormones, especially ABA and auxin, participate in signal transduction pathways during the response to abiotic stress [38]. ABA is an important signaling molecule in the plant adaptive response to cold stress [39]. In response to cold conditions, ABA-responsive element binding factor (ABF) can activate the expression of downstream genes [40]. Five ABF genes (four upregulated and one downregulated) were identified, and this finding was consistent with the increase in ABA content from N_1_ to CA_3_. Apart from the genes related to signaling, ABA-IMPORTING TRANSPORTER 1 (AIT1), an ABA importer gene, involved in ABA biosynthesis [41] was also identified. In this study, two AIT1 genes were identified, and expression levels of both were upregulated at CA_3_ indicating that ABA transport occurred actively under cold acclimation conditions. This finding was consistent with the variation in ABA content.

Studies suggest that auxin may lead to the rapid expression of certain genes related to cold tolerance and auxin metabolism is also part of the plant cold-resistance regulation system [42]. Numerous genes in the ‘auxin-activated signaling pathway’ and ‘response to auxin’ were detected in this study. Previous studies have shown that the protein serine/threonine kinase PINOID (PID) [43], an early auxin-inducible gene, regulates PIN localization on cellular membranes and thus regulates polar auxin transport [43, 44]. Currently, studies on PID genes mainly focus on many plants such as *Arabidopsis* [45], tobacco [46], cucumber [47], and rice [48], whereas studies on woody perennials have rarely been reported because most researchers select leaves or whole seedlings for investigation in experiments. In this study, shoot apexes (leaf bud) were used as they are considered the main existing organ for their vital role in survival during natural overwintering, and two PID genes (both with downregulated expression levels) were identified. The results confirmed that auxin plays an important role in cold response mediation in *M. denudata* and will thus be the focus of our future research.

### Carbohydrate metabolism in response to cold acclimation

Soluble sugars, constituting an important class of carbohydrates, can mitigate the damage caused by cold stress to plants by decreasing the freezing point of the cell, thus preventing membrane injury, and increasing the stability of the cell structure [49–51]. Cold acclimation promotes the development of cold resistance, and plants with robust cold resistance mechanisms tend to have higher soluble sugar content [52, 53]. In this study, soluble sugar content increased with an increase in cold stimulus. The rate of increase from N_1_ to CA_2_ was faster than that from CA_2_ to CA_3_, which indicated that soluble sugar and soluble protein accumulated more actively from N_1_ to CA_2_, to prevent the infliction of damage attributable to cold conditions. Meanwhile at CA_3_, the contents of soluble sugar and soluble protein remained at higher levels supporting the fact that *M. denudata* persisted at the cold-acclimation stage at CA_3_.

In addition to phenotypic data, the KEGG analysis helped identify 307 regulated genes in the ‘carbon metabolism’ pathway, 250 regulated genes in the ‘starch and sucrose metabolism’ pathway, 222 regulated genes in the ‘protein processing in endoplasmic reticulum’ pathway, and 141 regulated genes in the ‘amino sugar and nucleotide sugar metabolism’ pathway (Table 4 and Additional file 8: Fig. S2). This demonstrates that carbohydrate metabolism plays an important role in cold acclimation.

**Table 4:** Categorization of *M. denudata* unigenes to KEGG biochemical pathways

KEGG categories	Unigene -number	Ratio of no.	Pathway
Ribosome	315	3.35%	ko03010
Carbon metabolism	307	3.26%	ko01200
Biosynthesis of amino acids	292	3.11%	ko01230
Spliceosome	284	3.02%	ko03040
Plant hormone signal transduction	251	2.67%	ko04075
Endocytosis	251	2.67%	ko04144
Starch and sucrose metabolism	250	2.66%	ko00500
Protein processing in endoplasmic reticulum	222	2.36%	ko04141
Oxidative phosphorylation	218	2.32%	ko00190
Purine metabolism	202	2.15%	ko00230
RNA transport	198	2.11%	ko03013
Pyrimidine metabolism	166	1.77%	ko00240
Phenylpropanoid biosynthesis	166	1.77%	ko00940
mRNA surveillance pathway	159	1.69%	ko03015
PI3K-Akt signaling pathway	159	1.69%	ko04151
Ubiquitin mediated proteolysis	158	1.68%	ko04120
Glycolysis / Gluconeogenesis	154	1.64%	ko00010
Cell cycle	150	1.60%	ko04110
Amino sugar and nucleotide sugar metabolism	141	1.50%	ko00520
RNA degradation	138	1.47%	ko03018
Pyruvate metabolism	126	1.34%	ko00620
Glycerophospholipid metabolism	122	1.30%	ko00564
AMPK signaling pathway	121	1.29%	ko04152
Cell cycle - yeast	118	1.25%	ko04111
Oocyte meiosis	109	1.16%	ko04114
Peroxisome	107	1.14%	ko04146
Fatty acid metabolism	106	1.13%	ko01212
Cysteine and methionine metabolism	105	1.12%	ko00270
Phagosome	105	1.12%	ko04145
Photosynthesis	104	1.11%	ko00195
Ras signaling pathway	102	1.08%	ko04014
Ribosome biogenesis in eukaryotes	101	1.07%	ko03008
Regulation of actin cytoskeleton	100	1.06%	ko04810
Others	6414	68.21%	

Sucrose synthase (SuS) and sucrose phosphate synthase (SPS) are close related to sucrose metabolism and cold tolerance development [54, 55]. Expression of SuS and SPS is induced to exhibit responses to cold stress via regulation of sucrose metabolism [56]. In *Brassica oleracea* L*.* [57] and *Triticum aestivum* L*.* [58], the expression of SuS is induced by cold stress. In the transcriptome of *M. denudata*, seven genes (three upregulated and four downregulated) encoded SuS and one gene (upregulated) encoded SPS; most genes were expressed from N_1_ to CA_2_. Expression of four genes related to SuS were downregulated by low temperature. These results were not consistent with those reported by previous studies that explained the mechanisms by which SuS and SPS were involved in sucrose metabolism, and elucidated the manner in which sucrose metabolism influenced cold acclimation responses. These discrepancies warrant further study.

Similarly, trehalose and raffinose are important in sugar metabolism during cold acclimation [59, 60], and eight trehalose-phosphate phosphatases (four upregulated and four downregulated) and five raffinose synthases (three upregulated and two downregulated) were identified. These genes did not increase one-sidedly, which indicated that they played a limited role in the development of cold resistance in *M. denudata*. These synthases were found to mostly function at the cold acclimation stage from N_1_ to CA_2_; therefore, sugar metabolism might play a major role in the earlier stage of cold acclimation. Previous studies have identified Sugars Will Eventually Be Exported Transporters (SWEETs) as being involved in sugar transport and output [61–63]. In addition to the above-mentioned results, seven SWEET genes we also identified, and of these five SWEET genes were downregulated under cold acclimation conditions, including one *SWEET16* gene. *SWEET16* can catalyze the transport glucose and sucrose, and its expression is downregulated by low temperature in *Arabidopsis* and tea (*Camellia sinensis*). This indicated that under cold acclimation conditions, less amount of sugar was exported and more amount of sugar remained in the plants, and that carbohydrate metabolism contributed to cold tolerance development, a result consistent with those reported by previous studies.

### Cold-related TFs expressed in response to cold acclimation

To develop adaptation and resistance mechanisms to different stresses, plants correspondingly form self-suited regulatory systems, in which transcriptional regulation plays an important role. TFs play a key role in the environmental stress response network by binding to cis-acting elements in the promoter region and by regulating the expression of a series of genes [64].

AP2/ERF is a plant-specific gene family including four subfamilies, namely AP2, RAV, DREB and ERF. Among the AP2/ERF family, DREB, and ERF are closely related to exhibition of the plant abiotic stress response [65]. In this study, six CBF DEGs and 27 ERF DEGs were identified, which might serve as candidate genes for further study on the cold response mechanisms in *M. denudata*. In the DREB1 subgroup, DREB1A/CBF3, DREB1B/CBF1, and DREB1C/CBF2 can be induced under cold stress and the overexpression of DREB1s/CBFs significantly improves tolerance under abiotic stress [66–68]. *PdCBF2* increased in close correlation with cold acclimation in buds of almond (*P. dulcis*) [69]. In the six CBF DEGs, expression levels of four genes (two *CBF3*, one *CBF4* and one *CBF1*) were continuously upregulated during cold acclimation, while expression levels of two *CBF1* genes (CL64792Contig1 and CL63168Contig1) were downregulated at CA_2_ and then upregulated at CA_3_. In *Arabidopsis*, overexpression of *AtCBF1* induces COR genes and enhances cold tolerance [70]. These results further prove that CA_3_ is an important stage for the strengthening of cold tolerance. Whether the two *CBF1* genes in *M. denudata* possess specific functions warrants further study.

The bHLH gene family [71] plays an important role in the regulation of plant development and stress response [72]. In this study, 52 bHLH (10 upregulated and 42 downregulated) were identified. In a previous study, *ICE1* (inducer of CBF expression), a bHLH transcription factor, could specifically encode a MYC-like bHLH transcriptional activator that bound to the promoters of *CBF3* at low temperatures to induce the expression of *CBF3* [73, 74]. *ICE1* has been shown to positively regulate cold tolerance [74]. In this study, two genes encoding ICE (CL32817Contig1 and CL35094Contig1) did not upregulate after cold acclimation according to a previous study in *Arabidopsis* [74, 75], while the expression of *CBF3* was upregulated. Therefore, understanding the role of *ICE1* in the development of cold resistance and the causes of *CBF3* upregulation in *M. denudata* merits further study.

In this study, AP2/ERF and bHLH families were identified as two highly active groups in response to cold acclimation, indicating that *M. denudata* might improve cold tolerance through signaling pathways and transcription regulated by AP2/ERF and bHLH protein. However, further study is warranted to understand the relationships among TFs to enhance cold resistance mechanisms in *M. denudata*.

### Antioxidation mechanisms in response to cold acclimation

Reactive oxygen species (ROS) are rapidly accumulated when plants counter different abiotic stresses. ROS can act as a signal switch in response to environmental stress but can also cause oxidative damage to plants [76]. In this study, the MDA content increased with the extension of cold acclimation, indicating the occurrence of ROS accumulation. Superoxide dismutase (SOD), ascorbate peroxidase (APX), and catalase (CAT) are crucial enzymes that counter ROS, and play an important role in conferring antioxidant protection to plants [77, 78]. Their significant upregulation can effectively reduce oxidative damage inflicted by ROS and enhance low-temperature tolerance in plants. The expression increased with decreased temperature. Under cold stress conditions, SOD, CAT, and APX genes in *M. denudata* were significantly and differentially expressed, and the up-regulated genes played an important role. The expression levels of CL27100Contig1 (SOD) and CL38755Contig1 (SOD) were up-regulated, whereas those of CL11817Contig1 (CAT) and CL34986Contig1 (APX) were downregulated. This resulted in no significant difference in the MDA content between CA_2_ and CA_3_.

## Conclusion

**Fig. 6:**
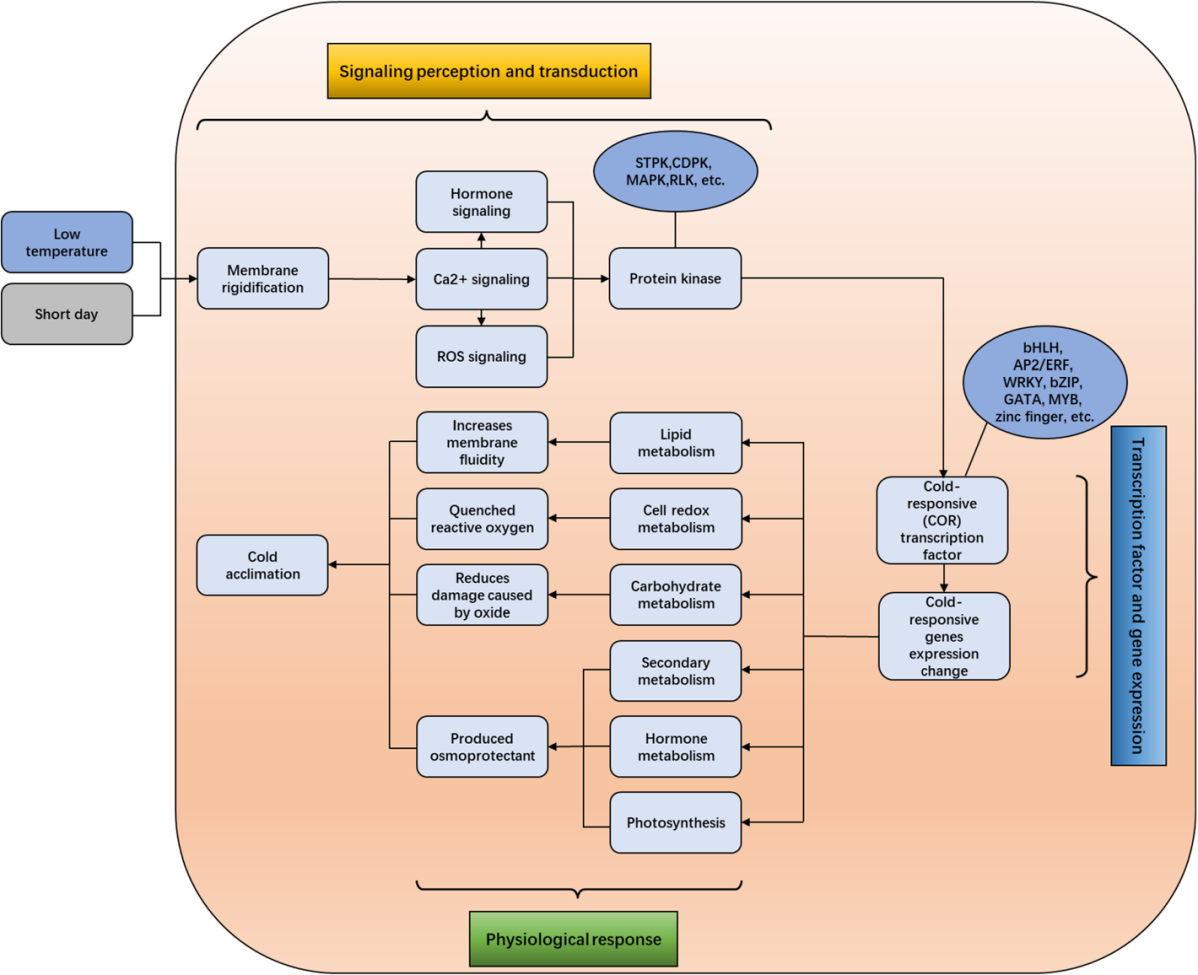
A hypothetical responsive diagram in *M. denudata* buds during cold acclimation

In this study, using de novo sequencing, a *M. denudata* dataset containing 74,503 unigenes was constructed to observe the dynamic changes of gene expression under different cold acclimation stages. A considerable number of DEGs were identified under cold acclimation conditions, especially those involved in cold signal perception and transmission, hormone regulation, TFs related to cold regulation and adaptation, and those involved in the elimination of ROS in antioxidation mechanisms. Based on the transcriptomic data, a responsive diagram of low temperature and SD in *M. denudata* has been hypothetically summarized in Fig. 6.

Transcriptome differences were compared based on different temperatures; comparisons with the control (N_1_ vs. CA_3_, N_1_ vs. CA_3_, and CA_2_ vs. CA_3_) helped identify the genes responsive to cold stress. Among the three pairwise comparisons, there were 357 upregulated and 235 downregulated transcripts between the cold-stress and control samples. Based on annotation analysis, pathways especially proline and hormones-related involve in cold acclimation and cold tolerance enhancement in *M. denudata*. From N_1_ to CA_2_, *M. denudata* experienced the most important cold acclimation stage, and cold tolerance was further strengthened at CA_3_. The transcriptome expression profiling of *M. denudata* may facilitate our understanding of the molecular regulation mechanism related to cold acclimation in *M. denudata*. This study also provides insights to improve cold resistance in species that are sensitive to cold injury in the Magnoliaceae family.

## Methods

### Plant materials and cold acclimation treatments

**Fig. 7:**
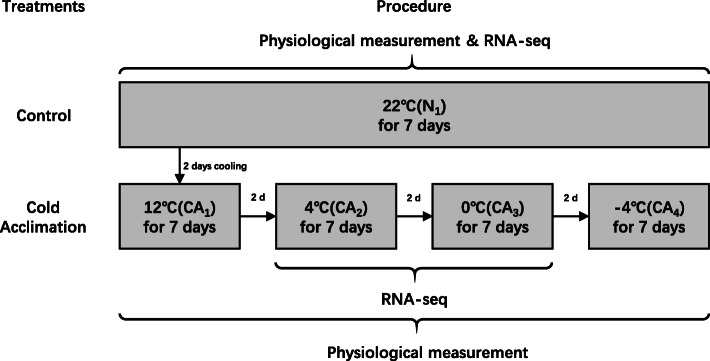
Experimental design of cold acclimation in *M. denudata***.** A room temperature of 22 °C was served as the control (N_1_) and then *M. denudata* buds were treated at low temperature of 12 °C (CA_1_), 4 °C (CA_2_), 0 °C (CA_3_), and − 4 °C (CA_4_) for seven sequential days. Two days were left for cooling slowly in temperatures incubator between two groups

*M. denudata* seeds were obtained from a single tree and after conducting sterilization and seeding, the seedlings of *M.denudata* were cultured in single pots in growth chambers with a temperature of 25 ± 1.0 °C (day) / 22 ± 1.0 °C (night), a photoperiod of 12-h light/12-h dark and 65% relative humidity in February 2017. The three apical buds of a six-month-old seedling per pot were selected randomly and used for experiments during the whole cold acclimation treatment in September 2017 (autumn). The experiment included five temperatures for analysis. A room temperature of 22 °C in a low-temperature incubator (3 M, USA) served as the control (N_1_). The samples in other groups of *M. denudata* buds were treated for seven days sequentially in low-temperatures incubators at the following four different experimental temperatures: low temperature of 12 °C (CA_1_), 4 °C (CA_2_), 0 °C (CA_3_), and − 4 °C (CA_4_). Two days were left for cooling slowly in temperatures incubator between two groups. Additionally, three groups (CA_2_, CA_3_, and N_1_) of living *M. denudata* buds were subjected to seven days of cold acclimation treatment for RNA-seq. The long-term cold acclimation (22 °C to − 4 °C) of *M. denudata* was also compared using physiological and biochemistry experiments (Fig. 7).

### Assay of physiological index contents

Physiological index contents of the leaf buds were determined by performing the following methods. The LT50 and proline content were determined according to the method described by Duan et al. and Bates et al. [79, 80]. The ABA content was estimated by performing high-performance liquid chromatography according to the method described by Xiao et al. [81]. Soluble sugar and protein contents were determined using the method described by Yang et al. [82]. The MDA content was assessed according to the thiobarbituric acid method. Each experiment included three biological and technical replicates and each biological replicate included three plants.

### RNA isolation

Total RNA isolation from the leaf buds of N_1_, CA_2_, and CA_3_ groups was performed using the mirVana™ miRNA Isolation Kit (Ambion-1561) according to the manufacturer’s instructions. RNA samples were assessed using the Agilent Bioanalyzer 2100 (Agilent) and were quantified using the NanoDrop-2000 (Thermo Scientific). After removing the genomic DNA using DNase I (Takara), the high-quality RNA samples were used to construct the sequencing library. Each experiment included three biological replicates.

### cDNA library construction and sequencing

Total RNA was extracted using the mirVana miRNA Isolation Kit (Ambion) following the manufacturer’s instructions. RNA integrity was evaluated using the Agilent 2100 Bioanalyzer (Agilent). The libraries were constructed using TruSeq Stranded mRNA LTSample Prep Kit (Illumina) according to the manufacturer’s protocol. Then these libraries were sequenced on the Illumina sequencing platform (HiSeqTM 2500) and 125 bp/150 bp paired-end reads were generated. Each experiment included three biological replicates.

### De novo assembly and sequence annotation

Raw data (raw reads) were processed using Trimmomatic to control the quality and remove the connector [83]. Reads containing ploy-N and low quality reads were removed to obtain clean reads. After removing the adaptor and low quality sequences, the clean high-quality reads were assembled into expressed sequence tag clusters (contigs) and assembled into transcripts de novo using Trinity [84] (vesion: trinityrnaseq_r20131110) in paired-end method. The longest transcript was selected as a unigene according to the sequence similarity and length for subsequent analysis.

The function of the unigenes was annotated by conducting alignment of the unigenes with the NCBI non-redundant (NR), SwissProt, and Clusters of orthologous groups for eukaryotic complete genomes (KOG) databases using BLASTx [85] with a threshold E-value of 10^− 5^.The proteins with the highest hits to the unigenes were used to assign functional annotations. Based on the annotation in SwissProt, Swissprot ID was mapped to the GO term to obtain the GO annotation of the unigene. The unigenes were mapped to the KEGG database [86] to annotate their potential metabolic pathways.

### Analysis and functional enrichment of DEGs

FPKM [87] and read counts of each unigene were calculated using bowtie2 [88] and eXpress [89]. Unigenes were standardized by estimateSizeFactors. *P*-values and foldchange of unigenes based on the analysis of different groups were calculated by nbinomTest from DESeq [90]. The data on DEGs in each treatment group were then filtered based on the threshold *p*-value < 0.05 and |log2FoldChange| > 2. GO enrichment and KEGG pathway enrichment analysis of DEGs were conducted using R, according to the hypergeometric distribution.

### TFs analysis

TFs were predicted based on CDS predicted protein sequences. Plant TFs domains were obtained from HMM search (http://plntfdb.bio.uni-potsdam.de/v3.0/) and the unigenes were classified according to gene family information.

### qRT-PCR analysis of gene expression

To validate the accuracy of the RNA-seq results, 12 DEGs were selected for qRT-PCR validation and UBQ [91] was used as a reference gene for analysis. The purified RNA samples were reverse-transcribed to cDNA using 2 μg of RNA in a 20 μl reaction system with the 5X All-In-One RT MasterMix with AccuRT (ABM, USA). The specific qRT-PCR primers were designed using Beacon Designer 7. qRT-PCR was conducted using the StepOnePlus™ Real-Time PCR System (Applied Biosystems, USA) and the final reaction contained 5 μl TB Green Premix Ex Taq (Tli RNaseH Plus) (2X), 0.2 μl ROX Reference Dye (50X) (Takara, Japan), 0.2 μl upstream primer, 0.2 μl downstream primer, 1.0 μL cDNA, and 3.4 μl ddH_2_O in a final volume of 10 μl. The primer sequences used in qRT-PCR are listed in Additional file 6 (Table S6). Each sample included three biological and technical replicates.

### Statistical analysis

The data were analyzed by one-way ANOVA followed by least significant difference test (*p* value < 0.05) using SPSS 22.0 software (IBM Corporation, USA). Graphs were constructed by SigmaPlot version 10 (Systat Software, USA) and R Project (R Foundation for Statistical Computing, Austria).

## Supplementary Information

**Additional file 1: Table S1**. Gene Ontology (GO) analysis of the differentially expressed genes (DEGs) for *M. denudata* buds in response to cold acclimation.

**Additional file 2: Table S2**. Kyoto Encyclopedia of Genes and Genomes (KEGG) analysis of differentially expressed genes (DEGs) for *M. denudata* buds in response to cold acclimation.

**Additional file 3: Table S3**. Differentially expressed transcription factors (TFs) of *M. denudata* buds in response to cold acclimation.

**Additional file 4: Table S4**. Differentially expressed protein kinases (PKs) of *M. denudata* buds in response to cold acclimation.

**Additional file 5: Table S5**. Differentially expressed genes (DEGs) related hormone metabolism and biosynthesis.

**Additional file 6: Table S6**. qRT-PCR primer information.

**Additional file 7: Fig. S1**. KEGG map about arginine and proline metabolism and arginine and proline metabolism compared with N_1_.

**Additional file 8: Fig. S2**. KEGG map about starch and sucrose metabolism compared with N_1_.

## Data Availability

The data sets used and analyzed during the study are available from the in the SRA database of NCBI (https://www.ncbi.nlm.nih.gov/bioproject/PRJNA683971).

## Abbreviations

ABA: Abscisic Acid
AP2/ERF: APETALA2/ETHYLENE RESPONSE FACTOR
APX: ascorbate peroxidase
bHLH: Helix–loop–helix
bZIP: Basic leucine zipper
CAT: catalase
CBF: C-repeat binding factor
CBLs: Calcineurin B-like protein
CDPKs: Calcium-dependent protein kinases
CIPKs: CBL-interacting protein kinases
DEGs: Differentially expressed genes
eggNOG: The evolutionary genealogy of genes: Nonsupervised Orthologous Groups database
FPKM: Fragments per kb per million fragments
GO: Gene ontology
ICE: inducer of CBF expression
LT50: Median Lethal Temperature
KEGG: Kyoto encyclopedia of genes and genomes
KOG: Eukaryotic ortholog group
MDA: Malondialdehyde
Nr: the NCBI non-redundant
Pfam: The Protein families database
qRT-PCR: Real-time quantitative reverse transcription PCR
ROS: Reactive oxygen species
TFs: Transcription factors
SOD: Superoxide dismutase
