# Supplementary material for: Physiological and transcriptome analysis of Magnolia denudata leaf buds during long-term cold acclimation
Source: BMC Plant Biol. 2021 Oct 8;21:460. doi: 10.1186/s12870-021-03181-5 (PMC8501692; doi:10.1186/s12870-021-03181-5)
Supplement: Supplementary file 7 — Additional file 7: Fig. S1. KEGG map about arginine and proline metabolism and arginine and proline metabolism compared with N1. [file 12870_2021_3181_MOESM7_ESM.docx]

(A)


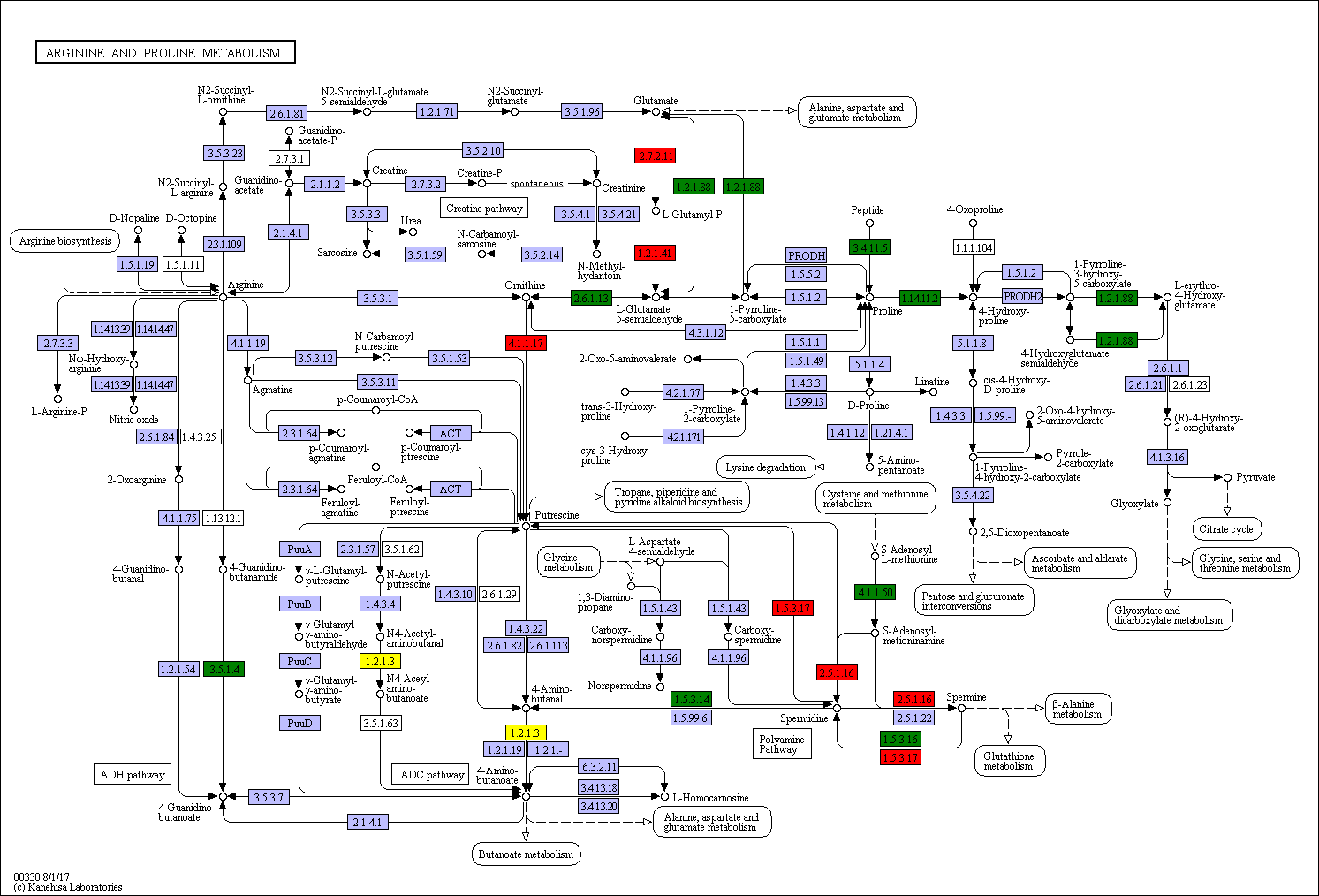


(B)


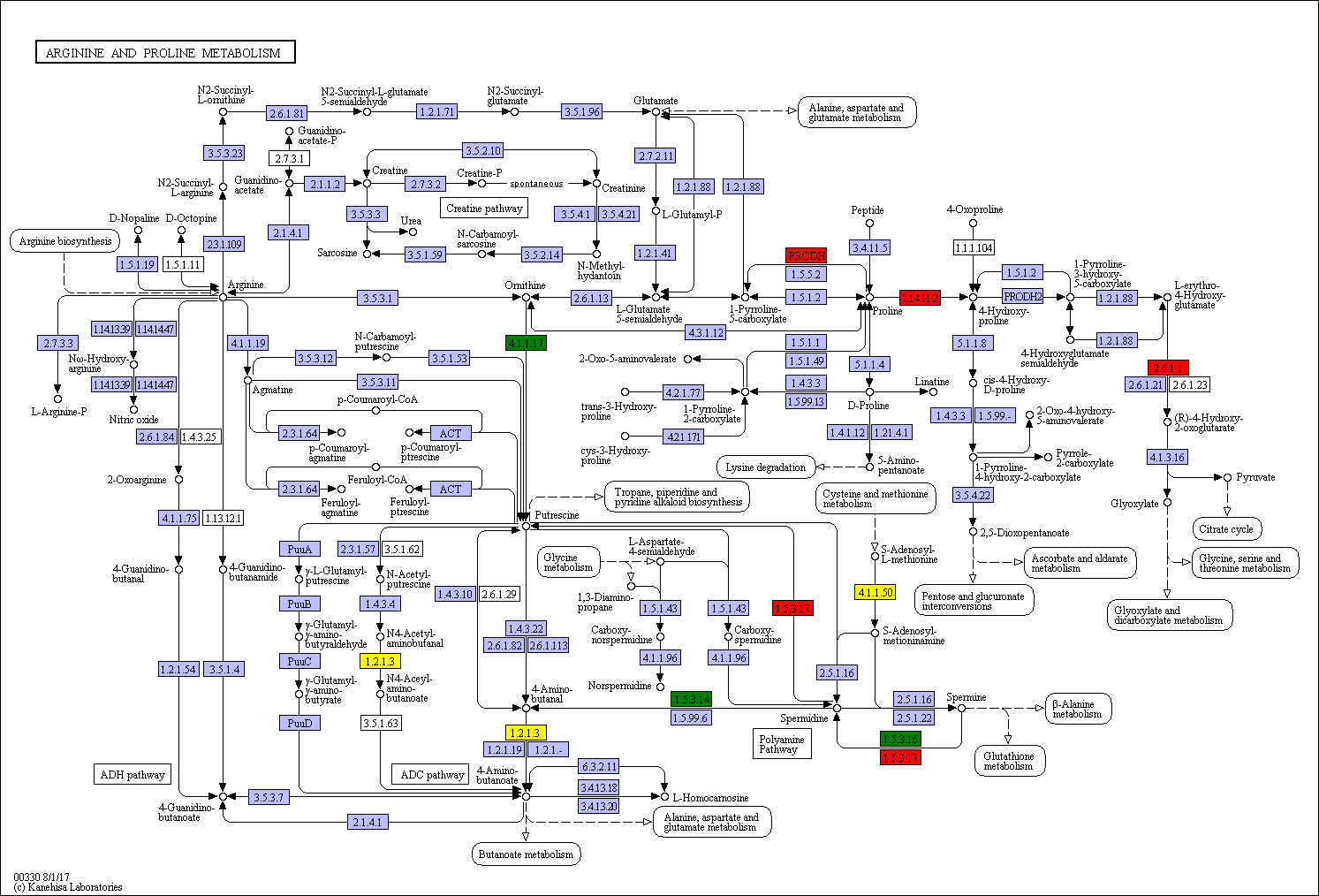


**Additional file 7：Figure S1.** **KEGG map about arginine and proline metabolism compared with N_1_. Red indicates significantly upregulated; green indicates significantly downregulated; yellow indicates proteins encoded by both up- and downregulated genes; purple indicates no significantly changed.**

**(A)** CA_2_ vs. N_1_; **(B)** CA_3_ vs. N_1_
